# Mitochondrial Dysfunction in Aged Macrophages and Lung during Primary *Streptococcus pneumoniae* Infection is Improved with Pirfenidone

**DOI:** 10.1038/s41598-018-37438-1

**Published:** 2019-01-30

**Authors:** Maria Plataki, Soo Jung Cho, Rebecca M. Harris, Hua-Rong Huang, Ha Seon Yun, Kristen T. Schiffer, Heather W. Stout-Delgado

**Affiliations:** 1 Department of Medicine, Pulmonary and Critical Care, Weill Cornell Medicine, New York, NY USA; 2 Department of Respiratory and Critical Care Medicine, Shanghai Pulmonary Hospital, Tongji University School of Medicine, Shanghai, China

## Abstract

Pneumococcal infections remain a leading cause of death in older adults, with the most serious cases occurring in persons ≥65 years of age. There is an urgent need to investigate molecular pathways underlying these impairments and devise new therapeutics to modulate innate immunity. The goal of our current study is to understand the impact of chronological aging on mitochondrial function in response to *Streptococcus pneumoniae*, a causative agent of bacterial pneumonia. Using chronologically aged murine models, our findings demonstrate that decreased ATP production is associated with dysregulated mitochondrial complex expression, enhanced oxidative stress, diminished antioxidant responses, and decreased numbers of healthy mitochondria in aged adult macrophages and lung in response to *S*. *pneumoniae*. Pre-treatment of aged macrophages with pirfenidone, an anti-fibrotic drug with antioxidant and anti-inflammatory properties, improved mitochondrial function and decreased cellular oxidative stress responses. *In vivo* administration of pirfenidone decreased superoxide formation, increased healthy mitochondria number, improved ATP production, and decreased inflammatory cell recruitment and pulmonary oedema in aged mouse lung during infection. Taken together, our data shed light on the susceptibility of older persons to *S*. *pneumoniae* and provide a possible therapeutic to improve mitochondrial responses in this population.

## Introduction

*Streptococcus pneumoniae* (*S*. *pneumoniae*) is the most common cause of community-acquired pneumonia and other types of infections, such as sepsis and meningitis, in the elderly (>65 years)^1,2^. Pneumococcal infections are responsible for significant morbidity and mortality in this age population^1,2^. Due to increased prevalence of comorbidities and dysregulated immune system responsiveness, pneumococcal pneumonia in the elderly can quickly result in increased edema and bacteremia. While it has been well established that innate immune responses to *S*. *pneumoniae* are impaired with aging, there is a pressing need to understand the molecular mechanisms that underlie this dysregulation and develop cutting-edge therapies that specifically target and amplify innate immune responses in older persons^3–10^.

Mitochondria are dynamic organelles that play an essential role in bioenergetics, metabolism, programmed cell death, and modulation of innate immune responses^11^. Mitochondrial oxidative metabolism and reactive oxygen species (ROS) production is important for mediating innate antibacterial signaling and bactericidal activity^12,13^. Mitochondria produce ATP by oxidative phosphorylation, synthesize and catabolize metabolites, and generate/detoxify ROS appropriately in response to cellular energy demands. Five mitochondrial protein complexes (complex I, II, III, IV, and V) play a key role in oxidative phosphorylation to create ATP. During the process of oxidative phosphorylation, these mitochondrial complexes, through a series of chemical reactions create an unequal electrical charge on either side of the inner mitochondrial membrane, which drives the production of ATP. Alterations in mitochondrial complex function can propel a vicious cycle in which an elevation of calcium levels, oxidative stress, and decreased ATP synthesis can occur^14^. Excessive or sustained mitochondrial stress can lead to disrupted mitochondrial membrane potential (ΔΨm), increased ROS production, and mitochondrial permeability transition pore (MPTP) formation. These detrimental changes in mitochondrial health eventually result in the uncoupling of the mitochondria, swelling of the matrix, mitochondrial rupturing, and cell death^15^. When MPTP opening is extensive, mitochondria become uncoupled and ATPase works in reverse mode, resulting in a significant reduction of ATP levels through ATP hydrolysis^16^. With reduced ATP levels, cells cannot maintain structural and functional integrity, including ion homeostasis, resulting in irreversible damage and cell death^16,17^.

Mitochondrial damage and abundance of mitochondrial damage-associated molecular patterns (DAMPs), such as mitochondrial DNA (mtDNA), has been linked to multiple innate signaling cascades and may contribute to overly heightened systemic inflammatory responses^18–20^. Recent work has illustrated that the pore forming toxin, pneumolysin, can directly induce mitochondrial dysfunction and release of mtDNA by human alveolar epithelial cells^21^. Enhanced bacterial toxin mediated alterations of mitochondrial function may underlie augmented tissue damage and elevated morbidity and mortality of older persons in response to *S*. *pneumoniae*.

A key feature of mitochondrial aging is the increased number of large deletions and somatic point mutations in mtDNA, which may be attributed to increasing levels of oxidative damage^22–30^. Despite the important role that mitochondria play in host responsiveness to pathogenic stimuli, very little is known about the impact of age-associated alterations in mitochondrial function on innate immune responses to *S*. *pneumoniae*, a causative agent of bacterial pneumonia. Our findings demonstrate that decreased ATP production is associated with dysregulated mitochondrial complex expression, enhanced oxidative stress, diminished antioxidant responses, and decreased numbers of healthy mitochondria in response to *S*. *pneumoniae*. Pirfenidone (5-methyl-1-phenyl-2-[1*H*]-pyridone) (PRF), an orally active small molecule with antioxidant, anti-inflammatory, and anti-fibrotic characteristics has recently been shown to reduce cytosolic mtDNA, improve ROS scavenging, and enhance electron transport chain function^31–33^. Our results show that pre-treatment with pirfenidone improved mitochondrial function, increased healthy mitochondria number, improved ATP production, and decreased inflammatory cell recruitment and pulmonary oedema in aged mouse lung during infection. Taken together, our data provide additional evidence as to why older persons are more susceptible to *S*. *pneumoniae* and suggest a possible therapeutic to improve mitochondrial responses in this population.

## Results

### Mitochondrial Membrane Potential (ΔΨm) Instability in Aged Lung during *S*. *pneumoniae* Infection

**Figure 1:**
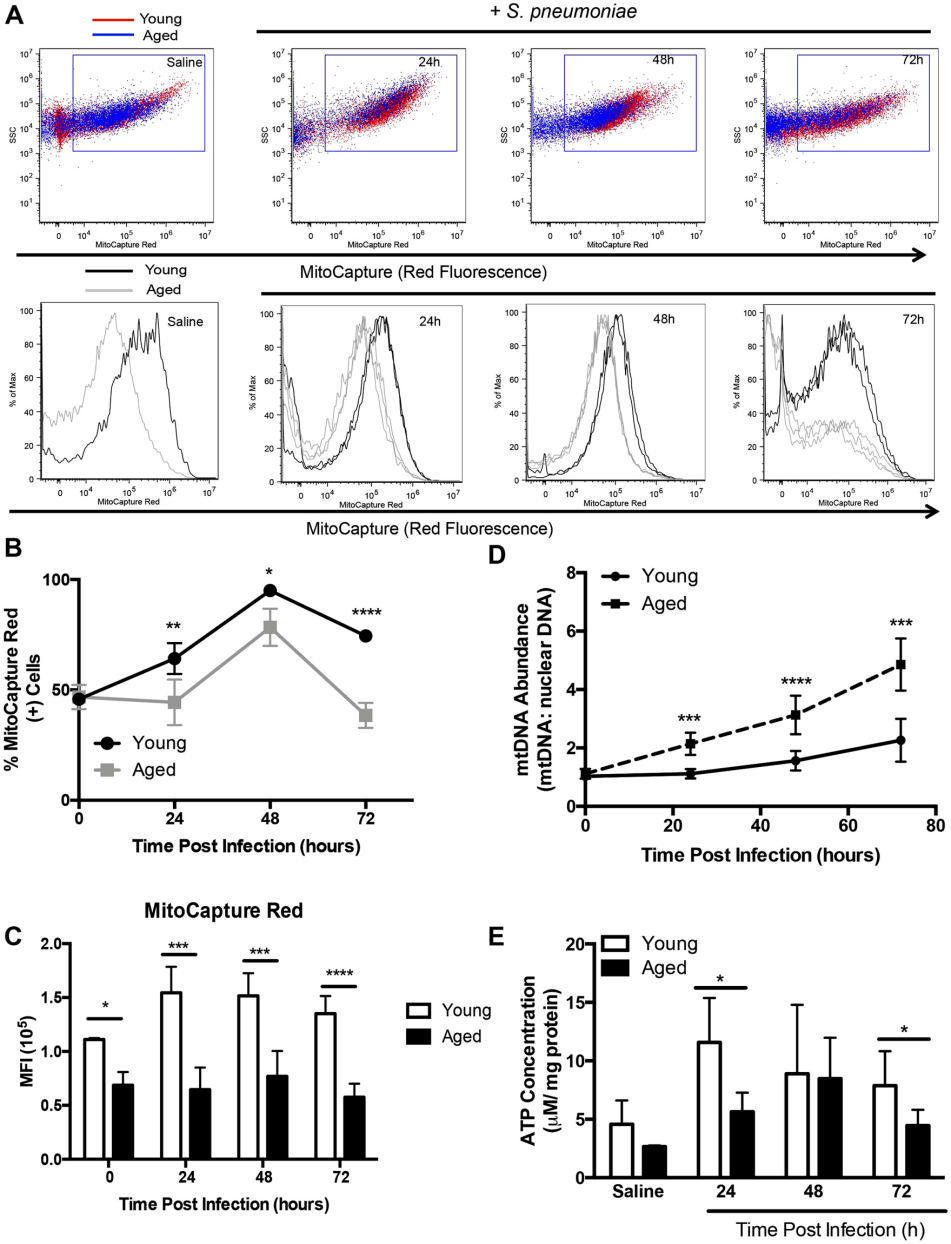
Mitochondrial Membrane Potential (ΔΨm) in Aged Lung Declines during *S*. *pneumoniae* Infection. Young (2 months) and aged (19 months) male and female BALB/c mice received 1 × 10^6^ CFU of *S*. *pneumoniae* (ATCC 6303) or saline via intranasal instillation. Lung tissue from young and aged uninfected and *S*. *pneumoniae* infected mice was collected at select time points post infection. (**A**) ΔΨm was examined in lung cells by assessing changes in MitoCapture red and green fluorescence. Dot plots: young (red cells) and aged (blue cells). Histograms: young (black line) and aged (grey line). For histograms, N = 3 are shown for the 24–72 hour time points. (**B**) % of MitoCapture red positive cells and (**C**) mean fluorescence intensity (MFI) was assessed for each sample (t-test: ^*^P < 0.05, ^**^P < 0.01, and ^****^P < 0.0001). (**D**) DNA was isolated from uninfected and *S*. *pneumoniae* infected lung tissues and the ratio of mtDNA to nuclear DNA levels were assessed (t-test: ^***^P < 0.001 and ^****^P < 0.0001). (**E**) ATP concentration in young and aged lung was assessed at baseline and in response to infection (t-test: ^*^P < 0.05). Similar results were obtained from at least two or more independent experiments with 10 mice per experiment. Data are expressed as the mean ± SD.

To examine the impact of chronological aging on mitochondrial function, we intranasally instilled young (2 months of age) and aged (19 months of age) mice with a highly virulent type 3 strain of *S*. *pneumoniae* commonly associated with increased relative risk of death in older persons^34^. We collected lung tissue and examined ΔΨm in single cell suspensions using MitoCapture apoptosis detection reagent. In healthy cells, MitoCapture dye accumulated and aggregated in the mitochondria, yielding a bright red fluorescence. In apoptotic cells, the MitoCapture dye, due to altered ΔΨm, was unable to aggregate in mitochondria and remains in a monomeric green fluorescing form within the cytoplasm. We examined MitoCapture fluorescence in lung in response to *S*. *pneumoniae*. Within the first 24–48 hours post infection, there was a disruption of the ΔΨm, as indicated by decreased MitoCapture red fluorescence in aged lung cells (Fig. 1A–C). By 72 hours of infection, ΔΨm was altered in aged lung as illustrated by a decrease in MitoCapture aggregates in mitochondria (Fig. 1A–C). As extensive ΔΨm instability can contribute to mtDNA abundance, we examined mtDNA expression in young and aged lung at select time points during infection^35,36^. While baseline mtDNA abundance was similar in young and aged lung, there was a significant increase in abundance in aged lung in response to *S*. *pneumoniae* (Fig. 1D). Extensive mitochondrial damage can result in decreased ATP production. In response to *S*. *pneumoniae*, ATP production was significantly decreased in aged lung at 24 and 72 hours post infection (Fig. 1E). Taken together, in response to *S*. *pneumoniae*, there was increased disruption of the ΔΨm, augmented mtDNA abundance, and decreased ATP production in aged lung.

### Disrupted ΔΨm in Aged Macrophages during *S. pneumoniae* Infection

**Figure 2:**
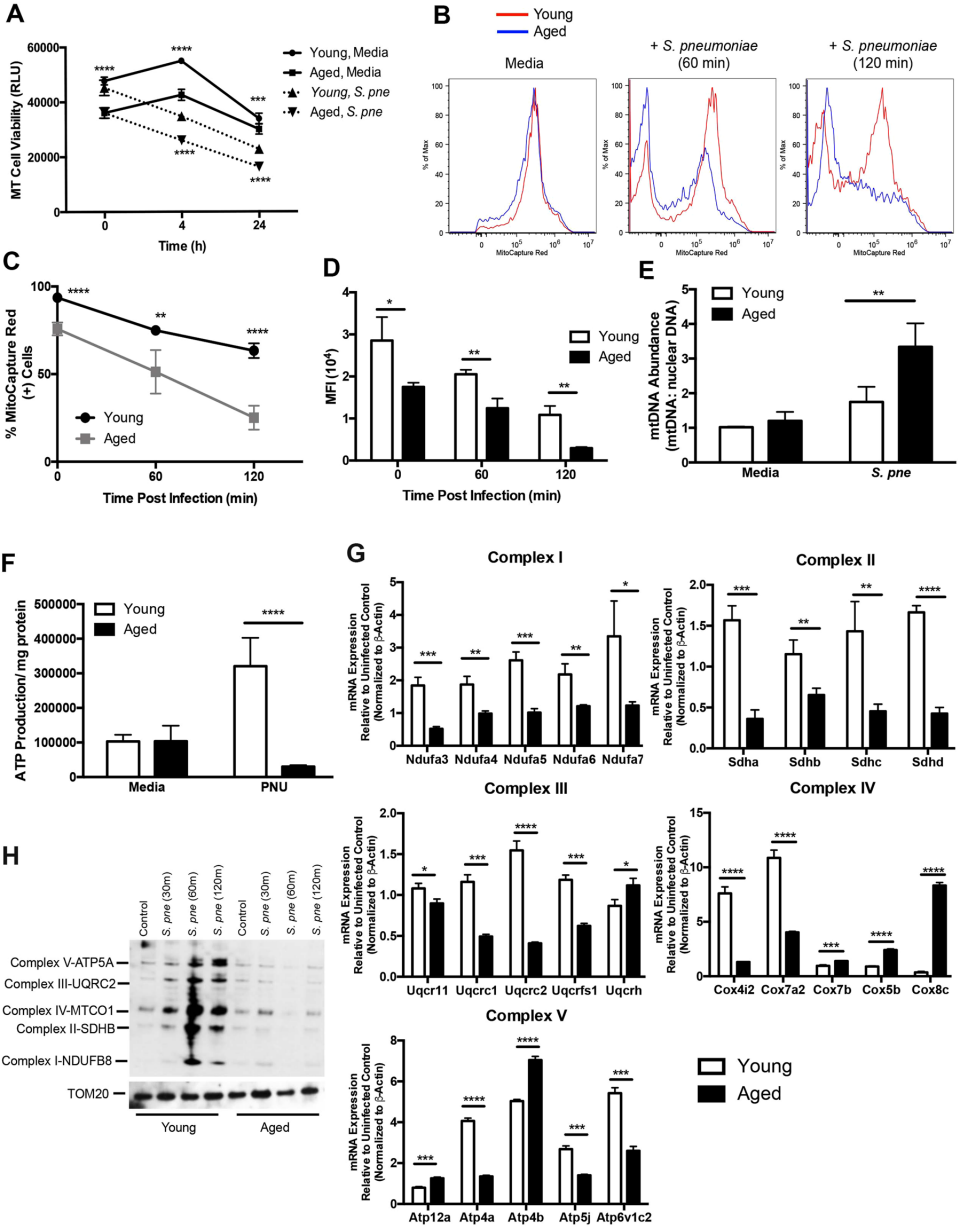
Mitochondrial Membrane Potential (ΔΨm) is disrupted in Aged Macrophages during *S*. *pneumoniae* Infection. Young and aged macrophages were cultured with *S*. *pneumoniae* (MOI = 25). (**A**) Cell viability was quantified at 4 and 24 hours post infection using the MT cell viability assay. Light production by metabolically active cells was proportional to the number of live cells in culture (t-test: ^***^P < 0.001 and ^****^P < 0.0001). (**B**) At 60 and 120 minutes post *S*. *pneumoniae* infection, cells were collected and changes in mitochondrial membrane potential (ΔΨm**)** were assessed by flow cytometric changes in MitoCapture red fluorescence. (**C**) % of MitoCapture red positive cells and (**D**) mean fluorescence intensity (MFI) was assessed for each sample (t-test: ^**^P < 0.01 and ^****^P < 0.0001). (**E**) DNA was isolated from uninfected and *S*. *pneumoniae* infected macrophages at 24 hours post infection and the ratio of mtDNA to nuclear DNA levels were assessed by real time PCR (t-test, ^**^P < 0.01). (**F**) ATP concentration in young and aged macrophages was assessed at baseline and in response to 4 hours of infection (t-test: ^****^P < 0.0001 and one-way ANOVA ^****^P < 0.0001). (**G**) mRNA was isolated from young and aged macrophages at select time points of *S*. *pneumoniae* infection and gene expression was analyzed by real time PCR using RT^2^ Profiler Assays (Mouse Mitochondrial Energy Metabolism PAMM-008ZA). Specific mRNA expression of genes associated with complex I (NDUF, NADH:Ubiquinone Oxioreductase), complex II (SDH, succinate dehydrogenase), complex III (UQCR, ubiquinol-cytochrome C reductase), complex IV (COX, cytochrome C oxidase), and complex V (ATP synthase) were quantified (t-test: ^*^P < 0.05, ^**^P < 0.01, ^***^P < 0.001, and ^****^P < 0.0001 and one-way ANOVA ^****^P < 0.0001). (**H**) Mitochondria were isolated from young and aged macrophages at select time points post infection and protein expression was assessed using the Total OXPHOS antibody cocktail. The blot was stripped and re-probed for TOM20 expression to confirm equivalent protein input between lanes. Full-length gels and densitometric analysis using Image J software are included in Supplemental Fig. 1 for comparison. Similar results were obtained from at least three independent experiments. Data are expressed as the mean ± SD.

As macrophages play an essential role in innate antibacterial immunity, we examined the impact of chronological aging on mitochondrial function in macrophages in response to *S*. *pneumoniae*. Using the MT Cell Viability Assay, we first investigated the impact of chronological aging on macrophage viability during infection. In healthy, viable cells, the viability substrate was reduced and a luminescent signal was generated. At baseline, reduction of the viability substrate was significantly decreased in aged macrophages (Fig. 2A). In response to *S*. *pneumoniae*, there was a decline in cell viability in both young and aged macrophages, with significantly lower levels of healthy aged macrophages (Fig. 2A). We evaluated if disruption of the ΔΨm, an early intracellular event during apoptosis, was elevated in aged macrophages. Decreased baseline ΔΨm in aged macrophages is further reduced in response to *S*. *pneumoniae* (Fig. 2B–D). As mitochondrial membrane instability can contribute to increased mtDNA expression, we quantified mtDNA abundance in young and aged macrophages during infection. Relative mtDNA abundance was significantly augmented in aged macrophages at 2 hours post *S*. *pneumoniae* infection (Fig. 2E). As changes in ATP production can be indicative of changes in mitochondrial function, we measured intracellular ATP production in young and aged macrophages. In response to infection, ATP production in aged macrophages was markedly reduced when compared to young macrophages (Fig. 2F). Alterations in mitochondrial structure and expression can contribute to changes in mitochondrial function. To determine if chronological aging contributed to changes in mitochondrial complex expression, we isolated RNA and protein from young and aged macrophages and examined changes in gene expression during infection. Mitochondrial oxidative phosphorylation is a major cellular source of ROS. Under specific metabolic or stress conditions, electrons can prematurely exit the mitochondrial respiratory chain, resulting in mitochondrial superoxide generation. Electron leakage occurs at complexes I, II, and III, with complexes I and III being the major sites of superoxide generation^37,38^. Complex I (NADH:Ubiquinone Oxioreductase, NDUF), complex II (succinate dehydrogenase, SDH), complex III (ubiquinol-cytochrome C reductase, UQCR), and complex IV (cytochrome C oxidase, COX) gene expression was heightened in both young and aged macrophages in response to *S*. *pneumoniae*, with highest levels of expression detected in young macrophages (Fig. 2G). Interestingly, in response to infection, there was differential mRNA expression of genes associated with formation of the complex V (ATP synthase), in both young and aged macrophages (Fig. 2G). To examine protein expression, we isolated mitochondria from young and aged macrophages in response to *S*. *pneumoniae* (Fig. 2H, Supplemental Fig. 1). When compared to young, expression of proteins associated with complex I–V was decreased in aged *S*. *pneumoniae* infected macrophages (Fig. 2H). In sum, decreased cell viability of aged macrophages in response to *S*. *pneumoniae* was associated with increased mtDNA abundance, decreased ATP production, alterations in ΔΨm, and decreased complex I–V gene expression.

### Pirfenidone Treatment Decreases Oxidative Stress in Aged Macrophages in Response to *S*. *pneumoniae*

**Figure 3:**
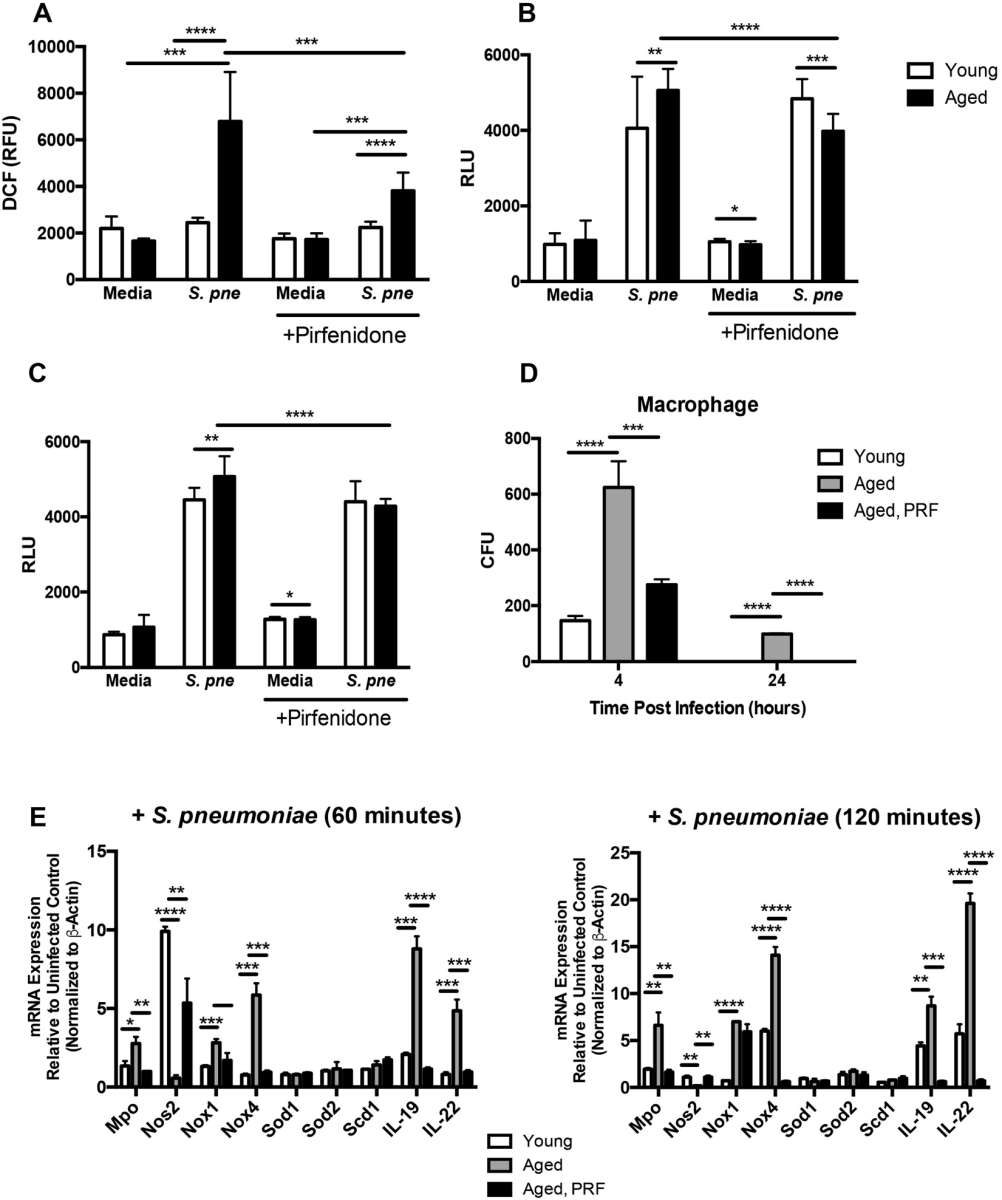
Pirfenidone Treatment Decreases Oxidative Stress in Aged Macrophages in Response to *S*. *pneumoniae*. Aged and/or young macrophages were cultured with media alone or media containing pirfenidone (500 μg/ml) for 24 hours prior to culture with *S*. *pneumoniae* (MOI = 25). (**A**) At 2 hours post *S*. *pneumoniae* infection, changes in cellular ROS levels were examined using H_2_DCFDA (10 μM) and changes in DCF fluorescence was assessed (t-test: ^***^P < 0.001 and ^****^P < 0.0001 and one-way ANOVA ^****^P < 0.0001). (**B**) Lytic (cellular derived) and (**C**) non-lytic (secreted into cell culture media) hydrogen peroxide generation was assessed using the ROS-Glo H_2_O_2_ (t-test: ^*^P < 0.05, ^**^P < 0.01, ^***^P < 0.001, and ^****^P < 0.0001 and one-way ANOVA ^****^P < 0.0001). (**D**) Bacterial titers were assessed by serial dilution of media collected at 4 and 24 hours (t-test: ^****^P < 0.0001, one-way ANOVA ^****^P < 0.0001). Representative blood agar plate images are included in Supplemental Fig. 2 for comparison. (**E**) mRNA was isolated from young and aged macrophages at select time points of *S*. *pneumoniae* infection and gene expression was analyzed by real time PCR using RT^2^ Profiler Assays (Mouse Oxidative Stress PAMM-065ZA). Specific mRNA expression of myeloperoxidase (MPO), nitric oxide synthase 2 (NOS2), NADPH oxidase (NOX) 1 and 4, superoxide dismutase (SOD) 1 and 2, stearoyl-coenzyme A desaturase 1 (SCD1), and interleukin (IL) 19 and 22 are illustrated (t-test: ^*^P < 0.05, ^**^P < 0.01, ^***^P < 0.001, and ^****^P < 0.0001 and one-way ANOVA ^****^P < 0.0001). Similar results were obtained from at least three independent experiments. Data are expressed as the mean ± SD.

Previous work has illustrated that pirfenidone can act as a scavenger of hydroxyl and superoxide anions and that treatment with pirfenidone can improve mitochondrial function^31,39^. We evaluated the impact of pirfenidone pre-treatment on ROS production in young and aged macrophages during *S*. *pneumoniae* infection. Cellular levels of ROS were assessed by changes in DCF fluorescence in untreated and pirfenidone treated macrophages. In response to *S*. *pneumoniae*, ROS production was heightened in aged macrophages (Fig. 3A). Pre-treatment with pirfenidone significantly reduced ROS production in *S*. *pneumoniae* infected aged macrophages (Fig. 3A).

During normal cellular metabolism, hydrogen peroxide (H_2_O_2_) formation increases and in response to certain physiologic and pathophysiologic conditions, can oxidatively modify cellular components. To assess cellular H_2_O_2_ formation, uninfected and infected cells were treated with a H_2_O_2_ substrate for two hours prior to cell lysis and addition of detection solution (Fig. 3B). As H_2_O_2_ is cell membrane permeable, in additional experiments H_2_O_2_ levels were quantified in cell culture media alone or media collected from uninfected and infected macrophages (Fig. 3C, Supplemental Fig. 2). In response to infection, there was an increase in cellular formation and release of H_2_O_2_ in both young and aged macrophages, with significantly higher levels detected in aged macrophages (Fig. 3B,C). We investigated the impact of pirfenidone on oxidant formation during infection. Pre-treatment with pirfenidone significantly decreased formation and release of H_2_O_2_ in aged *S*. *pneumoniae* infected macrophages that corresponded with improved bacterial clearance (Fig. 3B–D, Supplemental Fig. 2). As pirfenidone treatment did not significantly reduce ROS production or H_2_O_2_ formation in young macrophages, we focused on the impact of pirfenidone treatment on the expression of genes involved in ROS production and superoxide metabolism in aged macrophages. Specifically, we examined the expression of myeloperoxidase (MPO), nitric oxide synthase 2 (NOS2), NADPH oxidase (NOX) 1 and 4, superoxide dismutase (SOD) 1 and 2, stearoyl-coenzyme A desaturase 1 (SCD1), and interleukin (IL) 19 and 22. When compared to untreated aged macrophages, pre-treatment with pirfenidone reduced the expression of genes associated with ROS metabolism and oxidative stress responses during infection (Fig. 3E). Taken together, treatment of aged macrophages with pirfenidone reduced cellular ROS and H_2_0_2_ production, and reduced expression of genes associated with oxidative stress in response to *S*. *pneumoniae*.

### Pirfenidone Treatment Improves Mitochondrial Respiration and ATP Production in Aged Macrophages in Response to *S*. *pneumoniae*

**Figure 4:**
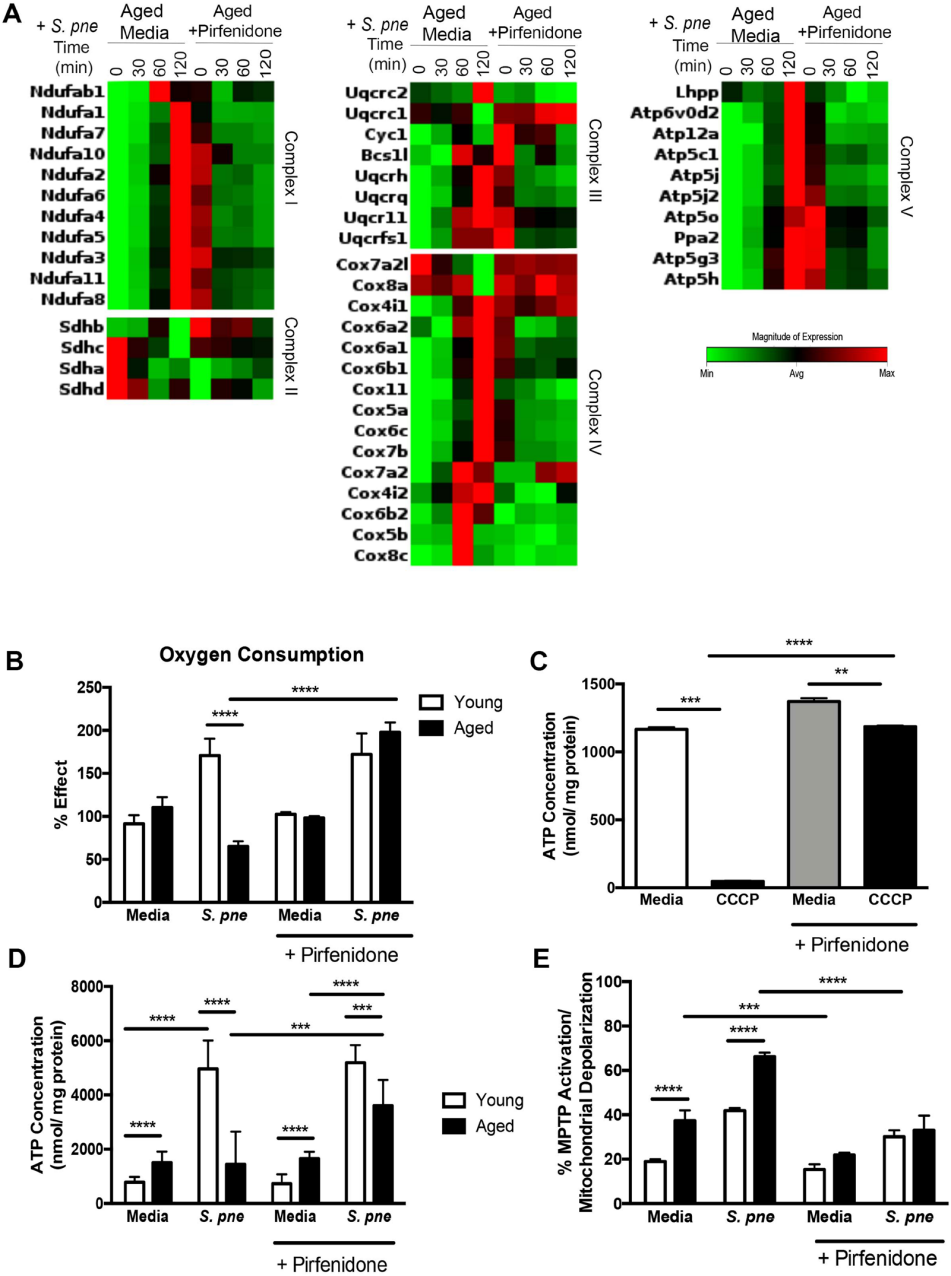
Pirfenidone Treatment Improves Mitochondrial Respiration and ATP Production by Aged Macrophages in Response to *S*. *pneumoniae*. Aged and/or young macrophages were cultured with media alone or media containing pirfenidone (500 μg/ml) for 24 hours prior to culture with *S*. *pneumoniae* (MOI = 25). (**A**) mRNA was isolated from untreated or pirfenidone treated aged macrophages at select time points of *S*. *pneumoniae* infection and gene expression was analyzed by real time PCR using RT^2^ Profiler Assays (Mouse Mitochondrial Energy Metabolism PAMM-008ZA, fold regulation values are listed in Table 1). (**B**) Mitochondrial respiration was assessed using the MITO-ID Extracellular O_2_ Sensor Kit. Pirfenidone treated cells were cultured with *S*. *pneumoniae* (2 hours) prior to addition of the MITO-ID Extracellular O_2_ probe. Results were calculated as % effect from age-matched media treated samples (t-test: ^****^P < 0.0001 and one-way ANOVA ^****^P < 0.0001). (**C**) ATP concentration in untreated and pirfenidone treated aged macrophages was evaluated in the presence or absence of CCCP (25μM) (t-test: ^**^P < 0.01, ^***^P < 0.001, and ^****^P < 0.0001). (**D**) ATP concentration in response to pirfenidone young and aged macrophages was examined at baseline and at 2 hours of infection (t-test: ^***^P < 0.001 and ^****^P < 0.0001 and one-way ANOVA ^****^P < 0.0001). (**E**) MPTP activation was measured in young and aged macrophages after 2 hours post *S. pneumoniae* infection (t-test: ^***^P < 0.001 and ^****^P < 0.0001 and one-way ANOVA ^****^P < 0.0001). Similar results were obtained from at least three independent experiments. Data are expressed as the mean ± SD.

Inhibition of the mitochondrial respiratory chain can contribute to significantly augmented levels of oxidative stress. We examined the impact of pirfenidone pre-treatment on the expression of complex I–V gene expression in aged macrophages. In response to pirfenidone, there was a dramatic increase in baseline expression of multiple complex I–V genes (Fig. 4A, Table 1). Pre-treatment of aged macrophages with pirfenidone resulted in improved regulation of complex I–V gene expression in response *S*. *pneumoniae* (Fig. 4A, Table 1). Oxygen consumption is essential for ATP production. Using a phosphorescent oxygen sensitive sensor, we investigated the impact of pirfenidone on cellular respiration and mitochondrial function. During infection, oxygen consumption in aged macrophages was markedly reduced in response to infection (Fig. 4B). In response to pirfenidone, there was a significant increase in mitochondrial respiration and oxygen consumption in aged, infected macrophages (Fig. 4B). To further these findings, we evaluated if pre-treatment with pirfenidone could improve mitochondrial respiratory chain function in aged macrophages. To this extent, we examined ATP production in untreated and pirfenidone pretreated aged macrophages. In response to carbonyl cyanide m-chloro phenyl hydrazine (CCCP), there was a significant decrease in ATP production by aged macrophages that was rescued by pirfenidone (Fig. 4C). We investigated the impact of pirfenidone on ATP production by infected macrophages. When compared to untreated macrophages, ATP production during infection was significantly increased in pirfenidone treated aged macrophages (Fig. 4D).

**Table 1:** Relative expression of Mitochondrial Energy Metabolism genes in aged macrophages at select time points post *S*. *pneumoniae* infection (represented in heat map, Fig. 4).

GENE SYMBOL	GENBANK	Aged, *S*. *pne* (30 min)		Aged, *S*. *pne* (60 min)		Aged, *S*. *pne* (120 min)	
		Expression Relative to Aged Control	Standard Deviation	Expression Relative to Aged Control	Standard Deviation	Expression Relative to Aged Control	Standard Deviation
Ndufab1	NM_028177	1.020	0.445	1.682	0.019	1.356	0.514
Ndufa4	NM_010886	1.580	0.420	3.580	0.241	7.160	0.363
Ndufa6	NM_025987	1.729	0.455	4.317	0.995	9.448	0.289
Ndufa5	NM_026614	1.580	0.521	3.732	0.558	7.464	0.064
Ndufa1	NM_019443	1.729	0.903	4.141	0.378	13.177	0.016
Ndufa7	NM_023202	1.591	0.638	4.028	0.299	9.714	0.069
Ndufa3	NM_025348	1.434	0.049	2.732	0.029	4.756	0.069
Ndufa11	NM_027244	1.376	0.175	2.378	0.013	4.287	0.090
Ndufa2	NM_010885	1.444	0.293	3.249	0.093	5.315	0.146
Ndufa8	NM_026703	1.444	0.781	3.182	0.076	5.618	0.150
Ndufa10	NM_024197	1.087	0.181	1.257	0.081	1.765	0.077
Sdhb	NM_023374	1.214	0.473	2.000	0.201	1.591	0.300
Sdhc	NM_025321	1.050	0.005	1.248	0.161	1.366	0.505
Sdha	NM_023281	0.877	0.122	0.871	0.454	1.149	0.813
Sdhd	NM_025848	1.079	0.237	1.094	0.495	1.537	0.385
Uqcrc1	NM_025407	1.035	0.359	1.474	0.038	0.790	0.875
Uqcrc2	NM_025899	1.094	0.394	1.283	0.345	2.603	0.018
Uqcrfs1	NM_025710	1.275	0.006	1.959	0.421	2.395	0.526
Uqcr11	NM_025710	1.537	0.267	2.848	0.301	3.837	0.540
Uqcrh	NM_025650	1.670	0.848	3.433	0.760	6.364	0.542
Uqcrq	NM_025641	1.659	0.492	4.141	0.857	8.754	0.015
Cyc1	NM_025567	1.310	1.000	1.945	0.146	1.919	0.0741
Bcs1l	NM_025784	1.087	0.255	2.129	0.035	2.204	0.745
Cox8a	NM_025352	1.050	0.634	1.516	0.291	0.642	0.012
Cox4i2	NM_007750	2.042	0.002	4.056	0.684	5.502	0.012
Cox6a2	NM_053091	0.435	0.503	3.138	0.012	4.595	0.012
Cox4i1	NM_009943	1.329	0.240	2.311	0.199	3.434	0.520
Cox11	NM_009941	1.189	0.329	2.085	0.464	3.531	0.014
Cox6a1	NM_199008	1.591	0.211	3.758	0.118	7.362	0.015
Cox5a	NM_007748	1.625	0.582	3.138	0.159	7.516	0.052
Cox6c	NM_007747	1.659	0.039	4.563	0.128	10.629	0.852
Cox6b2	NM_053071	1.916	0.190	5.736	0.091	5.205	0.022
Cox5b	NM_183405	1.366	0.225	7.516	2.465	2.014	0.022
Cox8c	NM_009942	2.462	0.620	25.992	3.471	7.728	0.520
Cox7a2l	NM_009187	0.908	0.015	0.908	0.154	0.808	0.0145
Cox6b1	NM_025628	1.919	0.012	5.736	0.867	5.205	0.0152
Cox7b	NM_025379	1.892	0.015	4.377	0.965	9.000	1.6845
Cox7a2	NM_009945	4.141	1.025	12.553	1.265	11.794	1.9852
Lhpp	NM_029609	0.841	0.540	0.908	0.380	1.505	0.490
Atp5o	NM_138597	1.356	1.490	2.219	1.410	3.031	1.250
Ppa2	NM_146141	1.424	1.440	2.219	1.900	3.458	1.090
Atp5g3	NM_175015	1.474	1.630	3.271	1.620	4.757	1.300
Atp5h	NM_027862	1.347	1.600	3.074	1.590	4.659	1.480
Atp6v0d2	NM_175406	1.404	0.850	2.173	0.910	4.199	0.900
Atp12a	NM_138652	0.986	0.520	3.837	1.480	8.398	1.730
Atp5c1	NM_020615	1.659	0.180	3.706	0.210	8.340	1.890
Atp5j	NM_016755	1.892	0.153	4.377	1.970	10.853	1.650
Atp5j2	NM_020582	1.580	1.890	3.630	1.810	7.413	1.650
**GENE SYMBOL**	**GENBANK**	**Expression Relative to Aged PRF Control**	**Standard Deviation**	**Expression Relative to Aged PRF Control**	**Standard Deviation**	**Expression Relative to Aged PRF Control**	**Standard Deviation**
Ndufab1	NM_028177	1.094	0.0146	1.301	0.8800	1.274	0.0540
Ndufa4	NM_010886	2.462	0.0152	2.828	0.9400	2.204	0.4453
Ndufa6	NM_025987	3.411	0.0215	3.227	0.8200	2.621	0.4500
Ndufa5	NM_026614	2.713	0.0652	2.789	0.7800	2.235	0.7845
Ndufa1	NM_019443	3.095	0.0457	3.117	0.5900	3.272	0.1450
Ndufa7	NM_023202	3.095	0.5412	3.117	0.4900	2.751	0.4530
Ndufa3	NM_025348	2.602	0.6345	2.585	0.2500	2.395	0.4786
Ndufa11	NM_027244	2.235	0.6845	2.099	0.0900	1.972	0.6835
Ndufa2	NM_010885	2.189	0.1460	2.346	0.3000	1.815	0.5431
Ndufa8	NM_026703	2.789	0.0477	2.497	0.4800	2.282	0.4200
Ndufa10	NM_024197	1.404	0.8754	1.189	0.9000	1.189	0.3451
Sdhb	NM_023374	2.099	0.5412	2.346	0.7300	1.753	0.3451
Sdhc	NM_025321	1.310	0.0254	1.376	0.8900	1.248	0.3785
Sdha	NM_023281	0.895	0.0140	1.014	0.6500	1.014	0.7451
Sdhd	NM_025848	1.257	0.2560	1.310	0.6500	1.064	0.2100
Uqcrc1	NM_025407	2.378	0.5412	2.462	0.6700	2.189	0.5341
Uqcrc2	NM_025899	1.275	0.6853	1.133	0.3700	1.007	0.0120
Uqcrfs1	NM_025710	1.635	0.0025	1.853	0.4500	1.670	0.0396
Uqcr11	NM_025710	2.378	0.0853	2.462	0.4300	2.189	0.0354
Uqcrh	NM_025650	2.445	0.0125	2.346	0.3100	1.840	0.3451
Uqcrq	NM_025641	2.949	0.0165	3.294	0.1200	2.585	0.0123
Cyc1	NM_025567	2.028	0.3541	2.250	0.1300	1.602	0.0134
Bcs1l	NM_025784	1.558	0.9400	1.932	0.7300	1.505	0.0435
Cox8a	NM_025352	1.357	0.0700	1.790	0.6500	1.444	0.1354
Cox4i2	NM_007750	0.864	0.6900	0.757	0.8400	2.479	0.3520
Cox6a2	NM_053091	0.852	0.8000	1.064	0.7200	1.537	0.0320
Cox4i1	NM_009943	2.282	0.6100	2.621	0.3500	2.585	0.3658
Cox11	NM_009941	1.803	0.5400	1.777	0.4400	1.474	0.8300
Cox6a1	NM_199008	2.809	0.4900	3.364	0.2400	3.053	0.2900
Cox5a	NM_007748	2.462	0.4400	2.621	0.5000	2.144	0.2700
Cox6c	NM_007747	3.031	0.6300	2.949	0.1100	2.462	0.0540
Cox6b2	NM_053071	1.945	0.6000	1.879	0.5700	1.853	0.1570
Cox5b	NM_183405	1.101	0.8500	0.758	0.1800	1.485	0.5400
Cox8c	NM_009942	2.027	0.5200	4.028	0.8700	3.272	0.7600
Cox7a2l	NM_009187	1.301	0.1800	1.366	0.1500	1.257	0.7700
Cox6b1	NM_025628	2.412	0.1500	2.445	0.6700	2.497	0.5100
Cox7b	NM_025379	2.828	0.8900	3.010	0.6800	2.395	0.6500
Cox7a2	NM_009945	3.053	0.4500	10.629	0.1500	10.778	0.0093
Lhpp	NM_029609	0.785	0.0300	0.598	0.5440	0.697	0.0814
Atp5o	NM_138597	2.158	0.5000	2.219	0.5437	1.790	0.0125
Ppa2	NM_146141	2.099	0.7300	2.173	0.0540	1.558	0.0200
Atp5g3	NM_175015	2.362	0.6800	2.532	0.6635	1.853	0.5421
Atp5h	NM_027862	2.313	0.5800	2.479	0.3393	2.114	0.9651
Atp6v0d2	NM_175406	1.239	0.7400	1.424	0.5985	1.238	0.6523
Atp12a	NM_138652	2.204	0.3000	2.313	0.6531	2.462	0.6130
Atp5c1	NM_020615	3.160	0.0400	3.458	0.9631	2.694	0.4610
Atp5j	NM_016755	3.117	0.2900	3.095	0.3268	2.362	0.4130
Atp5j2	NM_020582	2.732	0.7500	2.828	0.0635	2.346	0.2541

**Figure 5:**
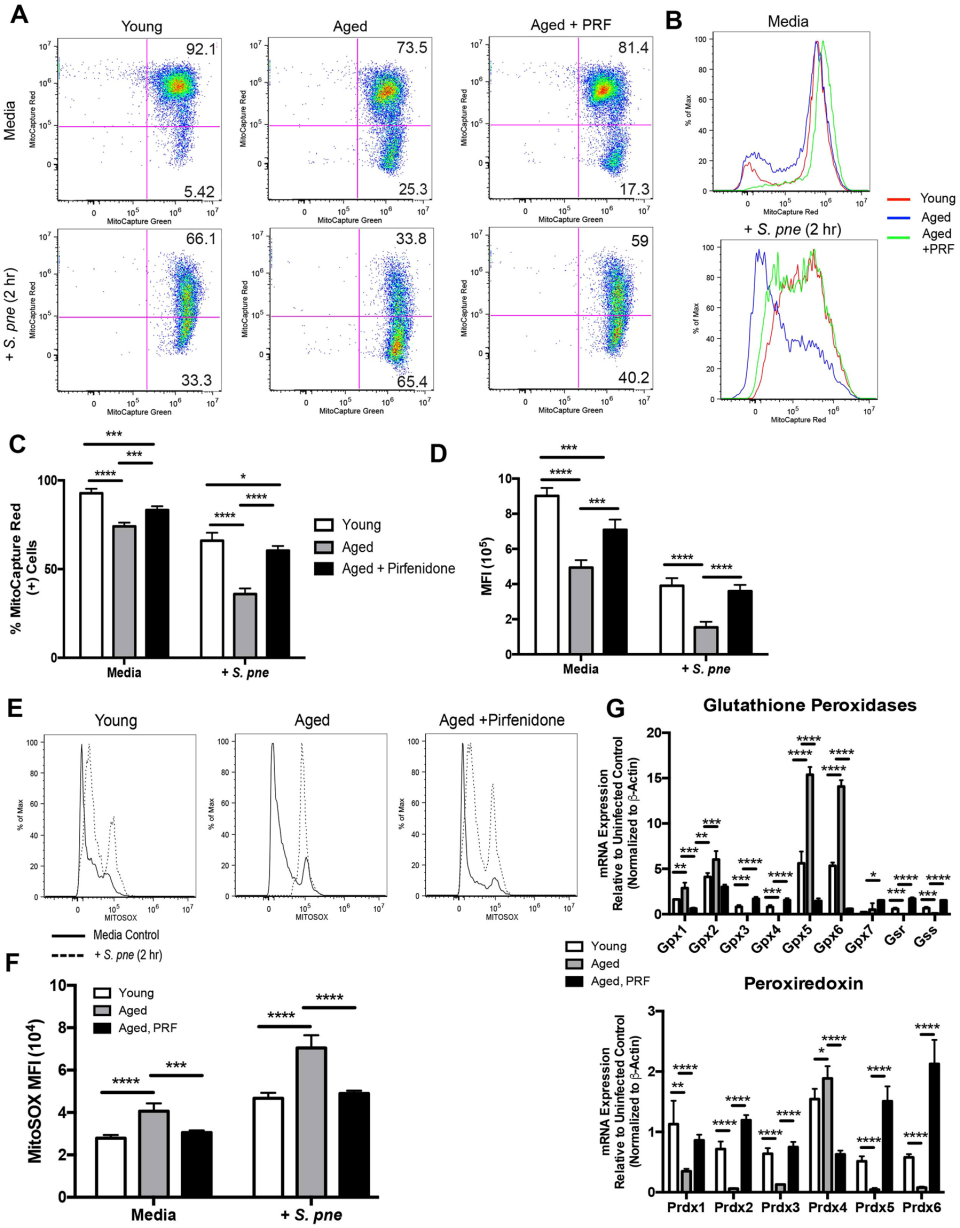
Pirfenidone Treatment Improves Mitochondrial Membrane Potential and Decreases Superoxide Production in Aged Macrophages during *S*. *pneumoniae* Infection. Young macrophages were cultured with media alone for 24 hours prior to culture with *S*. *pneumoniae* (MOI = 25). Aged macrophages were cultured with media alone or media containing pirfenidone (500 μg/ml) for 24 hours prior to culture with *S*. *pneumoniae* (MOI = 25). (**A**,**B**) ΔΨm was examined in young, aged, and pirfenidone treated aged macrophages by assessing changes in MitoCapture red and green fluorescence in response to 2 hours of *S*. *pneumoniae* infection. (**C**) % of MitoCapture red positive cells and (**D**) mean fluorescence intensity (MFI) was assessed for each sample (t-test: ^*^P < 0.05, ^**^P < 0.01, ^***^P < 0.001, and ^****^P < 0.0001 and one-way ANOVA ^****^P < 0.0001). (**E**) Superoxide formation in young, aged, and pirfenidone treated aged macrophages at 2 hours post *S*. *pneumoniae* infection was measured using MitoSOX (solid line: media control, dashed line: *S*. *pneumoniae* infected). (**F**) Mean fluorescence intensity (MFI) was assessed for each sample (t-test: ^***^P < 0.001 and ^****^P < 0.0001 and one-way ANOVA ^****^P < 0.0001). (**G**) mRNA was isolated from untreated and pirfenidone treated aged macrophages at select time points of *S*. *pneumoniae* infection. Gene expression was analyzed by real time PCR using RT^2^ Profiler Assays (Mouse Oxidative Stress PAMM-065ZA). Specific mRNA expression of glutathione peroxidase (GPX) 1–7, glutathione synthetase (GSS), glutathione reductase (GSR), and peroxiredoxin (PRDX) 1–6 are illustrated (t-test: ^*^P < 0.05, ^**^P < 0.01, ^***^P < 0.001, and ^****^P < 0.0001 and one-way ANOVA ^****^P < 0.0001). Similar results were obtained from at least three independent experiments. Data are expressed as the mean ± SD.

As sustained mitochondrial dysfunction can contribute to the opening of the MPTP, we examined if pirfenidone could modulate MPTP activation in aged macrophages during infection (Fig. 4E). In response to pirfenidone, there was a significant decrease in MPTP activation in infected aged macrophages (Fig. 4E). As MPTP activation and opening coincides with an increase in ΔΨm instability, we evaluated the impact of pirfenidone on the ΔΨm in aged macrophages. At baseline, decreased numbers of healthy mitochondria in aged macrophages was improved by pre-treatment with pirfenidone (Fig. 5A–D). In aged macrophages, there was a marked disruption of the ΔΨm in response to *S*. *pneumoniae*, which was restored with pirfenidone treatment (Fig. 5A–D). As oxidative stress was heightened in aged macrophages during infection, we examined if pirfenidone treatment could potentially reduce superoxide formation. Pre-treatment of aged macrophages with pirfenidone reduced superoxide formation during *S*. *pneumoniae* infection, as illustrated by decreased MitoSOX oxidation (Fig. 5E,F). We examined the potential mechanism by which pirfenidone might ameliorate oxidative stress in aged macrophages. We evaluated the expression of multiple antioxidant genes, such as glutathione peroxidases (GPX) 1–7, glutathione synthetase (GSS), glutathione reductase (GSR), and peroxiredoxins (PRDX) 1–6. In response to pirfenidone treatment, there was an increase in sustained expression of multiple GPX and PRDX genes during infection (Fig. 5G). In sum, pirfenidone treatment of aged macrophages improved mitochondrial respiration and ATP production, decreased MPTP activation, enhanced ΔΨm, and improved antioxidant gene expression during *S*. *pneumoniae* infection.

### Pirfenidone Treatment Improved ΔΨm and ATP Production in Aged Lung in Response to *S*. *pneumoniae*

**Figure 6:**
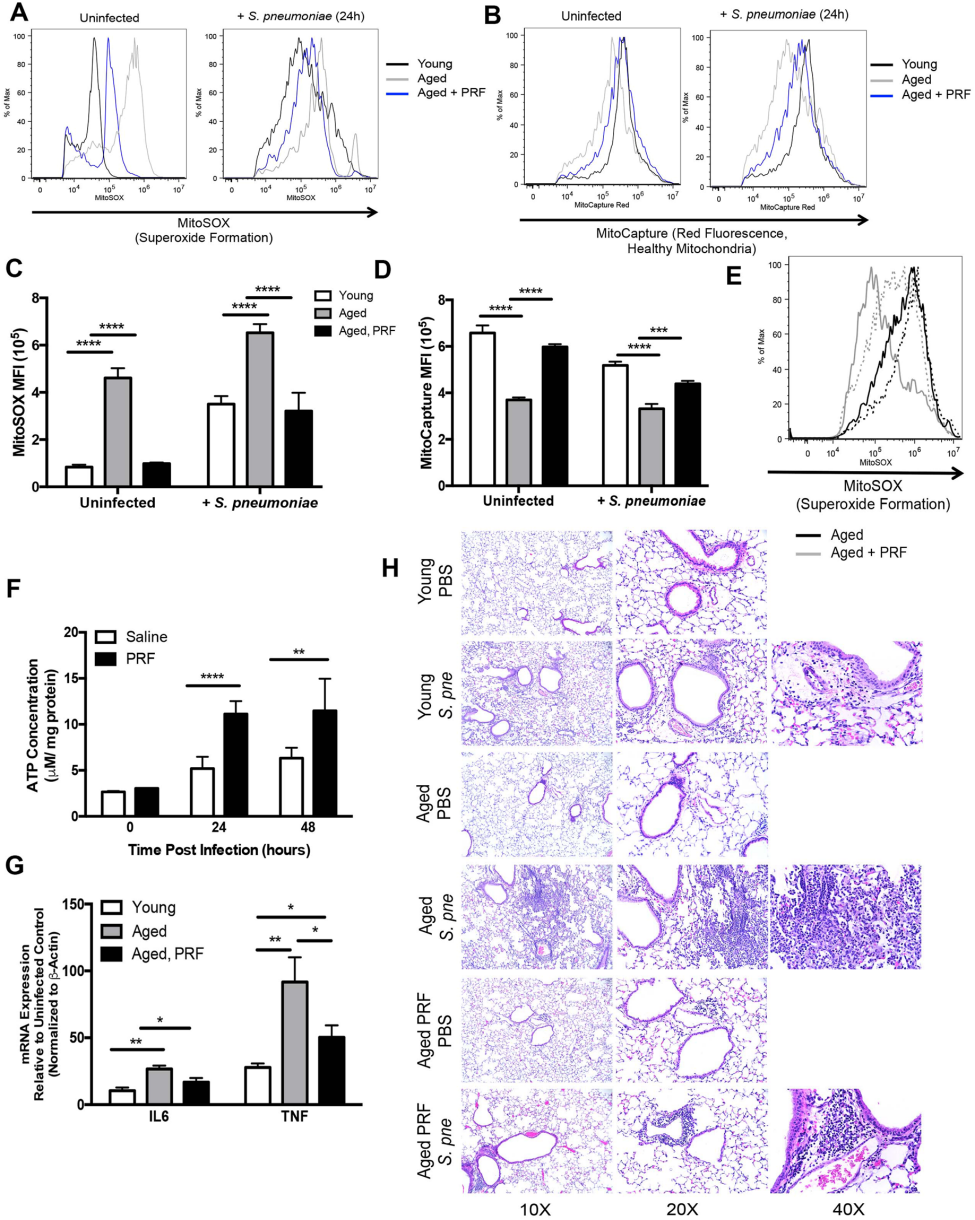
Pirfenidone Treatment Improves Antioxidant Responses in Aged Lung during *S*. *pneumoniae* Infection. Aged (19 months) male and female BALB/c mice received pirfenidone (280 mg/kg) intraperitoneally 24 hours prior and at the time of infection (1 × 10^6^ CFU of *S*. *pneumoniae* or saline via intranasal instillation). Lung tissue from aged untreated and pirfenidone treated mice collected was collected at 24 hours post *S. pneumoniae* infection. (**A**) Superoxide generation and ΔΨm was examined in aged control and pirfenidone treated lung tissues. Lung cells were collected at 24 hours post saline or *S*. *pneumoniae* instillation and evaluated by flow cytometry. Superoxide production was measured using MitoSOX. Young (black line), aged untreated (grey line), and aged pirfenidone treated (blue line). (**B**) Changes in MitoCapture red fluorescence was used to assess ΔΨm. Young (black line), aged untreated (grey line), and aged pirfenidone treated (blue line). (**C**,**D**) Mean fluorescence intensity (MFI) was assessed for each sample (t-test: ^***^P < 0.001 and ^****^P < 0.0001 and one-way ANOVA ^****^P < 0.0001). (**E**) Alveolar macrophages were isolated at 24 hours post saline or *S*. *pneumoniae* instillation. Superoxide formation was assessed by changes in MitoSOX fluorescence. Aged untreated (black line) and aged, pirfenidone treated (grey line). Saline instilled lung (solid line) and *S*. *pneumoniae* instilled (dotted line). (**F**) The impact of pirfenidone on baseline and *S*. *pneumoniae* induced ATP production in aged lung was assessed (t-test: ^**^P < 0.01 and ^****^P < 0.0001). (**G**) Lung tissue was isolated from young, aged, and aged pirfenidone treated mice at 24 hours post *S*. *pneumoniae* infection and IL6 and TNF mRNA expression was assessed by real time PCR (t-test: ^*^P < 0.05 and ^**^P < 0.01 and one-way ANOVA ^****^P < 0.0001). (**H**) H&E staining of lung tissue from control and *S*. *pneumoniae* infected mice collected at 24 hours post infection. Similar results were obtained from at least two or more independent experiments with 10 mice per experiment. Histology data is representative of data collected from 3 independent experiments with 10 mice per group. Data are expressed as the mean ± SD.

We evaluated the impact of pirfenidone treatment on mitochondrial function in aged lung during *S*. *pneumoniae*. For these experiments, aged mice were treated with PBS or pirfenidone (280 mg/kg) 24 hours prior and at the time of instillation. When compared to untreated aged mice, superoxide formation in infected lung was markedly decreased in response to pirfenidone (Fig. 6A,C). Similarly, when compared to PBS treated controls, pirfenidone treatment improved ΔΨm in aged lung during *S*. *pneumoniae* infection, as indicated by increased MitoCapture red fluorescence (Fig. 6B,D). To expand these findings, we next evaluated the impact of pirfenidone on superoxide formation in alveolar macrophages isolated from lung at 24 hours post saline or *S*. *pneumoniae* instillation. There was decreased superoxide formation in alveolar macrophages isolated from pirfenidone treated aged mice at baseline and in response to *S*. *pneumoniae* (Fig. 6E). We next investigated the impact of pirfenidone pre-treatment on ATP production in aged lung during infection. When compared to untreated mice, ATP production in aged lung in response to pirfenidone treatment was significantly increased at both the 24 and 48-hour time points post infection (Fig. 6F). We evaluated the impact of pirfenidone on inflammation and injury in aged lung during *S*. *pneumoniae* infection. When compared to young, there was a significant increase in immune cell infiltration and enhanced areas of inflammation and inflammatory cytokine expression in aged lung in response to infection (Fig. 6G,H). Treatment with pirfenidone improved bacterial clearance, reduced inflammation, pulmonary oedema, and inflammatory cytokine expression in aged lung during *S*. *pneumoniae* (Fig. 6G,H, Supplemental Fig. 3).

In contrast to young lung, there was decreased upregulation of multiple antioxidant genes in aged lung during infection (Supplemental Fig. 3). Based upon our *in vitro* findings, we examined if pirfenidone treatment might improve antioxidant gene expression in aged lung tissue at 24 hours post infection. Pre-treatment with pirfenidone rescued antioxidant signaling, resulting in heightened expression of glutathione peroxidases (GPX), peroxiredoxins (PRDX), and antioxidant genes in aged lung during infection (Supplemental Fig. 3). In sum, pirfenidone treatment enhanced ΔΨm, decreased superoxide formation, improved ATP production and antioxidant gene expression, as well as reduced inflammation in aged lung during *S*. *pneumoniae* infection.

## Discussion

The ΔΨm, which is generated by the proton pumps of complexes I, III, and IV, plays a key role in mitochondrial homeostasis and sustained changes may be deleterious and result in increased mitochondria dysfunction. In response to *S*. *pneumoniae*, mitochondria in aged lung retain the ability to respond to *S*. *pneumoniae*, as illustrated by increased in ΔΨm at 24–48 hours post infection. However, by 72 hours post infection, ΔΨm is diminished in aged lung. Decreased ΔΨm in aged lung correlated with decreased baseline and *S*. *pneumoniae* induced ATP production. A significant decrease in ATP levels, due to increased ATP usage and insufficient ATP generation, can result in enhanced acidification and degradation of cellular components. Treatment with pirfenidone, which improved ΔΨm, resulted in decreased superoxide formation and increased ATP production in lung during infection. It is therefore plausible, that age associated changes in ΔΨm contribute to diminished ATP homeostasis in aged lung in response to *S*. *pneumoniae*.

As most mitochondrial proteins are encoded in the nucleus, synthesized in the cytosol, and imported into the mitochondria, a decrease in ΔΨm may severely limit or suspend protein import. Despite decreased baseline expression, there was a time dependent increase in the transcription of complex I, III, IV and V genes in aged macrophages during infection. In response to pirfenidone, there was a marked improvement in ΔΨm that was associated with greater regulation and sustained expression of complex I–V genes in aged macrophages during infection. Treatment with pirfenidone and heightened antioxidant signaling may underlie increased complex expression and generation of ATP in aged macrophages in response to *S*. *pneumoniae*.

Instability in the ΔΨm can contribute to the opening of MPTP. Prolonged MPTP opening and increased depolarization can lead to decreased mitochondrial functionality and depending on the damage severity can induce cell death. When MPTP opening is extensive, mitochondria become uncoupled and ATPase works in reverse mode, resulting in a significant reduction of ATP levels through ATP hydrolysis. Recent work has illustrated that pneumolysin can reduce ΔΨm, with consecutive opening of the MPTP^21^. In agreement with these findings, there was an increase in MPTP activation and mtDNA abundance, with significantly higher levels in aged macrophages during *S*. *pneumoniae* infection. Results of our current study illustrate that in response to pirfenidone, there was a marked decrease in MPTP opening in aged macrophages at baseline as well as in response to *S*. *pneumoniae*. Diminishment of sustained MPTP activation in pirfenidone treated aged macrophages was associated with improved ATP production. Despite similar mtDNA abundance, it is possible that the mitochondrial membrane potential may be altered in aged macrophages. As the level of ΔΨm is kept relatively stable, it is plausible that decreased ΔΨm in aged mitochondria reflects a decrease in normal physiological activity. There was an increase in physiological activity during infection, as illustrated by enhanced ΔΨm in aged mitochondria. Significantly lower ATP levels in aged lung at 24 hours may reflect an energy supply/demand mismatch caused by insufficient ATP generation and proportionally increased use of ATP. By 48 hours post infection, mitochondrial membrane potential continues to rise in aged lung. This enhancement may contribute to increased ATP production at 48 hours, with levels similar between young and aged lung. By 72 hours post infection, there appears to be a significant loss of ΔΨm in aged lung. This decrease may be indicative of an inability to maintain ATP levels and decreased mitochondrial viability of aged lung cells.

While pirfenidone did not have a direct effect on *S*. *pneumoniae* growth, it might be possible that treatment during infection may counteract pneumolysin-mediated changes in ΔΨm. Pretreatment with pirfenidone resulted in a significant decrease in cellular ROS present in aged macrophages. Interestingly, pirfenidone has no significant effect on cellular ROS in young macrophages at baseline or in response to infection. It is plausible that overly heightened ROS production in aged macrophages may not be beneficial for cellular mediated clearance of *S*. *pneumoniae*. Taken together, a decrease in detrimental bacterial toxin induced mitochondrial dysfunction may underlie improved lung tissue pathology in aged pirfenidone treated mice in response to *S*. *pneumoniae*.

Mitochondrial oxidative phosphorylation is a major cellular source of ROS. Under specific metabolic or stress conditions, electrons can prematurely exit the mitochondrial respiratory chain, resulting in mitochondrial superoxide generation. Results of our current study illustrate increased cellular and mitochondrial ROS occurs in aged macrophages in response to *S*. *pneumoniae*. In addition, mitochondrial complex gene and protein expression are altered in aged macrophages in during *S*. *pneumoniae* infection. Complex I and III mRNA expression was increased in both young and aged macrophages, with highest levels of expression detectable in young macrophages at 2 hours post infection. In response to pirfenidone treatment, there was improved regulation and expression of complex I and III genes in aged macrophages during infection. Superoxide can be converted into H_2_O_2_ in the matrix by SOD2 or in the intermembrane space by SOD1^40^. In response to *S*. *pneumoniae*, there was augmented SOD1 and SOD2 mRNA expression in aged macrophages at 2 hours post infection. In the cytosol, superoxide is converted to H_2_O_2_ by SOD1. Examination of H_2_O_2_ production illustrated significantly higher levels of cellular and cytosolic H_2_O_2_ present in aged macrophages in response to *S*. *pneumoniae*. In response to pirfenidone, there was a time dependent decrease in SOD1 mRNA expression in aged macrophages that corresponds to significantly diminished levels of H_2_O_2_ present during *S*. *pneumoniae* infection. H_2_O_2_ can freely cross mitochondrial membranes or can be detoxified by mitochondrial antioxidant enzymes, such as glutathione peroxidase. Therefore, it is plausible that treatment with pirfenidone may function to detoxify enhanced superoxide and H_2_O_2_ production there thereby rescue mitochondrial signaling in aged macrophages and lung in response to *S*. *pneumoniae*.

In sum, results from our current study further illustrate the important role of mitochondria in modulating innate immune responses to *S*. *pneumoniae*. Impaired mitochondrial function, as illustrated by changes in ΔΨm, decreased ATP production, and increased superoxide formation in aged macrophages and lung contribute to increased lung pathology in response to infection. Treatment with pirfenidone improved mitochondrial function, decreased oxidative stress, and improved ATP production in aged, *S*. *pneumoniae* infected macrophages and lung.

## Materials and Methods

### Mice

Young adult (2 months) and aged adult (19 months) male and female BALB/c mice were purchased from the NIA rodent facility (Charles River Laboratories). Upon receipt, mice were handled under identical husbandry conditions and fed certified commercial feed. Body weights were measured daily and mice were humanely euthanized if they lost more than 15% of their starting body weight. The IACUC at Weill Cornell Medicine approved the use of animals in this study and methods were carried out in accordance with the relevant guidelines and regulations. No animals were used in the study if there was evidence of skin lesions, weight loss, or lymphadenopathy.

### Bacterial culture

*Streptococcus pneumoniae* (ATCC 6303, ATCC, Manassas, VA) was grown on 10% sheep blood agar plates (BD Biosciences, San Jose, CA) overnight. An inoculating loop of bacteria was cultured in Todd Hewitt broth (THB) containing 2% yeast extract for 4–6 hours (Sigma Aldrich). Bacterial cultures were centrifuged at 15,000 × g for 1 minute and resuspended in PBS. Colony forming units (CFU) were quantified by dilution of samples in THB and titers were determined by colony counts X dilution.

### *In vivo* procedures

#### S. pneumoniae infection

All mice were anesthetized with isoflurane (5% for induction and 2% for maintenance) prior to intranasal instillation with 1 × 10^6^ CFU of *S*. *pneumoniae* (50 μL volume in PBS).

#### Pirfenidone administration

Mice received a 100 μL volume of PBS (vehicle) or 280 mg/kg dose of pirfenidone (Sigma Aldrich) intraperitoneally 24 hours prior and at the time of infection. For all animal experiments, we used 10 mice per group and experiments were repeated at least three times.

### Primary bone marrow and alveolar macrophage isolation and cell culture

Bone marrow cells (BMCs) were prepared from the femurs and tibias of mice as previously described^9,41,42^. BMCs were treated with pirfenidone at time of plating (500 μg/ml, 24 h prior to stimulation). BMCs were cultured with media alone or media containing *S*. *pneumoniae* (MOI = 25). Alveolar macrophages were isolated from uninfected and *S*. *pneumoniae* infected mice (1 × 10^6^ CFU) at 24 h post instillation. Briefly, mice were lavaged with 5 × 1-ml of sterile PBS. Cells were collected and quantified. Cells were cultured in DMEM (10% FBS and 20% L929) for 4 hours prior to flow cytometric analysis. Pirfenidone was purchased from Sigma Aldrich and resuspended in DMSO per manufacturers recommendation.

### *In vitro* assays

Mitochondria were isolated from lung and macrophage cell cultures using the Mammalian Mitochondria Isolation Kit for Tissue and Cultured Cells (Catalog #: K288-50, Biovision, Milpitas, CA). Protein concentration of mitochondria was quantified by Bradford protein assay (Bio-Rad, Hercules, CA). *In vitro* assays: Cellular viability was evaluated by using the RealTime-Glo MT Cell Viability assay (Catalog #: G9711, Promega). Prior to infection with *S*. *pneumoniae*, MT viability substrate was added to cell culture media. Macrophages were cultured with *S*. *pneumoniae* for 2 hours, centrifuged at 500 g for 5 minutes prior to the start of each assay. ΔΨm was measured using the MitoCapture Fluorometric Kit (Catalog #: K250, Biovision). Briefly, cells were incubated with MitoCapture solution (1:1000 dilution) for 15 minutes at 37 °C. Mitochondrial superoxide generation was detected using MitoSOX red indicator (Catalog #: M36008, ThermoFisher Scientific). Cells were loaded with 1 ml of 5 μM MitoSOX and incubated for 10 minutes at 37 °C. Mitochondrial permeability transition pore (MPTP) activity in macrophages was assessed (Catalog #: K239, Biovision). Briefly, cells were incubated in 1:500 dilution of MPTP staining dye in the presence or absence of ionomycin or CoCl_2_ and incubated at 37 °C for 15 minutes. Cellular ROS was measured using H_2_DCFDA (Catalog #: D399, ThermoFisher Scientific). Young and aged macrophages were cultured with 10 μM H_2_DCFDA at 37 °C for 30 minutes. For all staining assays, cells were washed prior to assessment by flow cytometry and unstained, untreated, and carbonyl cyanide m-chloro phenyl hydrazine (CCCP: 25μM)-treated cells were used as controls. ATP levels in lung tissue samples were quantified using the ATP Determination Kit (Catalog #: A22066, ThermoFisher Scientific). ATP concentrations were determined by standard curve and normalized to sample protein concentration. Cellular ATP levels were assessed using the Mitochondrial ToxGlo Assay (Catalog #: G8000, Promega, Madison WI). ATP production was calculated and normalized to sample protein concentration. Lytic (cellular derived) and non-lytic (secreted into cell culture media) hydrogen peroxide generation was assessed using the ROS-Glo H_2_O_2_ Assay (Catalog#: G8820, Promega). Mitochondrial respiration was assessed using the MITO-ID Extracellular O_2_ Sensor Kit (Catalog #: ENZ-51045, ENZO Life Sciences). Pirfenidone treated cells were cultured with *S*. *pneumoniae* (2 hours) prior to addition of the MITO-ID Extracellular O_2_ probe. Results were calculated as % effect from age-matched media treated samples. Test compounds, oxamate (100 mmol), FCCP (0.2 μM), or antimycin (10 μM), were used to validate the assay. Flow cytometric analysis was performed using Flow Jo software (Tree Star Inc., Ashland, OR).

### DNA/RNA purification and real time PCR

DNA samples were extracted using the Qiagen DNeasy Blood & Tissue Kit (Germantown, MD). Samples were quantified and relative mtDNA expression (ratio of mtDNA to nuclear DNA) was assessed using the NovaQUANT Mouse Mitochondrial to Nuclear Ratio Kit (EMD MilliPore Sigma, Burlington, MA). RNA samples were extracted using the RNeasy Mini Kit (macrophage, Qiagen) or Trizol Plus RNA purification kit (lung tissue, ThermoFisher Scientific, Waltham, MA). Samples were quantified and A_260/280_ ratios were recorded. QuantiTect Primer Assays and RT^2^ Profiler^TM^ Assays (Mouse Mitochondrial Energy Metabolism PAMM-008ZA and Mouse Oxidative Stress PAMM-065ZA) were used to assess gene expression (Qiagen). All reactions were performed in triplicate. Relative levels of messenger RNA (mRNA) were calculated by the comparative cycle threshold method and either β−Actin or β2 M mRNA levels were used as the invariant control for each sample.

### Western blotting

Equal amounts of mitochondria (5–10 μg/lane) were loaded onto a 4–12% BIS-TRIS Bolt gel (ThermoFisher Scientific) and run at 200 V for 20 minutes. Protein was transferred to a nitrocellulose membrane using the iBlot Western blotting system (ThermoFisher Scientific). Immunodetection was performed using the Total OXPHOS Rodent WB Antibody Cocktail (1:500 dilution, catalog #: ab110413, Abcam, Cambridge, MA). Gels were treated with western blot stripping buffer and re-probed with αTOM20 (1:500 dilution, catalog #: sc-136211, Santa Cruz Biotechnology, Dallas, TX). Blots were quantified by using the ECL Western Blotting Analysis System (ThermoFisher Scientific).

### Histology

Mice were euthanized and right lung tissue was collected for downstream analysis. To maintain architecture, left lung was distended with 1% low melting agarose and placed into cold formalin^43^. Tissue samples were processed and H&E stained by the Translational Research Program at WCM Pathology and Laboratory of Medicine. Images were scanned using the EVOS FL Auto Imaging System (ThermoFisher Scientific).

### Statistical analysis

Survival analysis between groups was calculated using the Mantel Cox test. Comparison of groups was performed using a two-tailed t-test and comparisons between groups were verified by one-way ANOVA. All samples were independent and contained the same sample size for analysis. All data were analyzed using GraphPad Prism software (San Diego, CA). Statistical significance was considered by a ^*^P < 0.05, ^**^P < 0.01, ^***^P < 0.001, and ^****^P < 0.0001.

## Supplementary information

Supplemental Information

## Data Availability

Most data generated during this study are included in this published article and its Supplementary Information files. The datasets generated during and/or analyzed during the current study are available from the corresponding author on reasonable request.
