# Supplementary material for: Mitochondrial Dysfunction in Aged Macrophages and Lung during Primary Streptococcus pneumoniae Infection is Improved with Pirfenidone
Source: Sci Rep. 2019 Jan 30;9:971. doi: 10.1038/s41598-018-37438-1 (PMC6353918; doi:10.1038/s41598-018-37438-1)
Supplement: Supplementary file 1 — Supplemental Information [file 41598_2018_37438_MOESM1_ESM.pdf]

Supplemental Information:

Mitochondrial Dysfunction in Aged Macrophages and Lung during Primary *Streptococcus pneumoniae* Infection is Improved with Pirfenidone

Maria Plataki[1], Soo Jung Cho[1], Rebecca M. Harris[1], Hua-Rong Huang[1,2], Ha Seon Yun[1], Kristen T. Schiffer[1], and Heather W. Stout-Delgado[1]

1: Department of Medicine, Pulmonary and Critical Care, Weill Cornell Medicine, New York, NY

2: Department of Respiratory and Critical Care Medicine, Shanghai Pulmonary Hospital, Tongji University School of Medicine, Shanghai, China

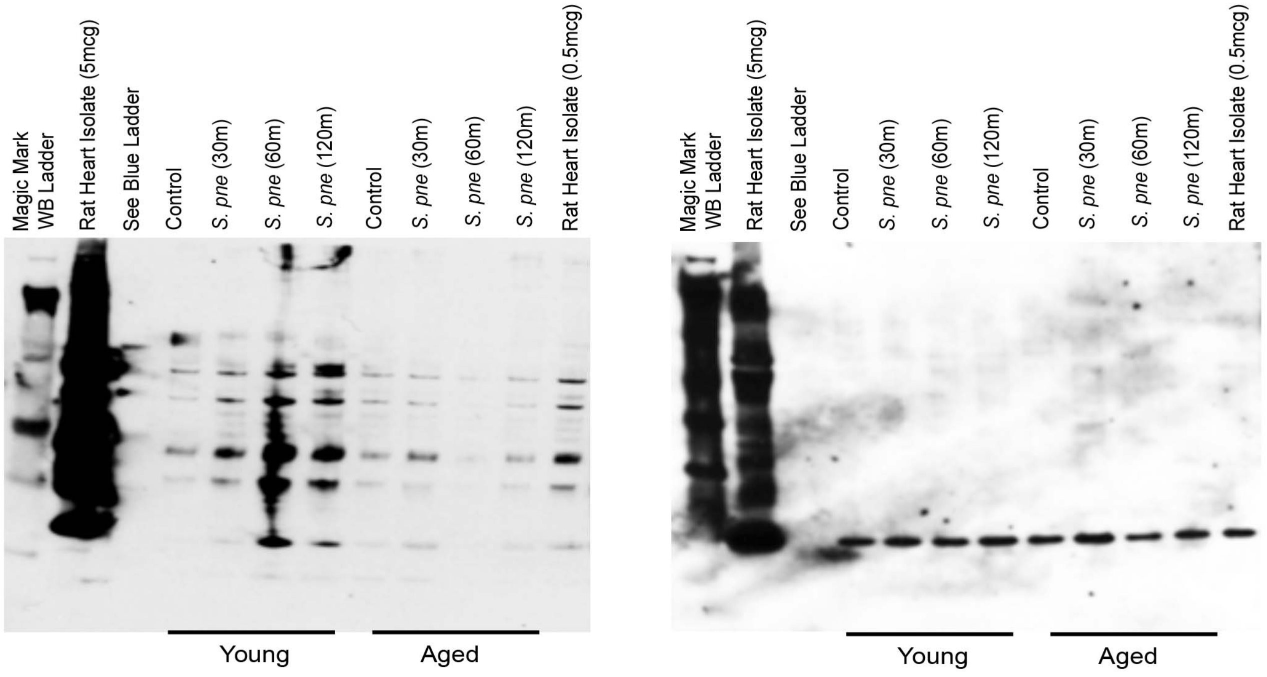

Uncropped
OXPHOS Western Blot

Uncropped
TOM20 Western Blot

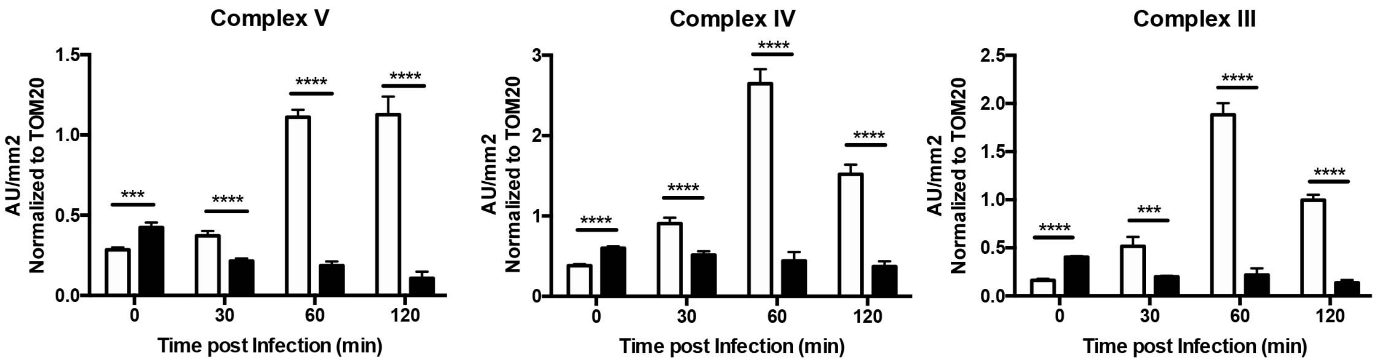

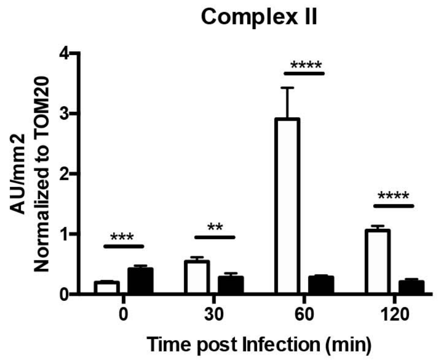

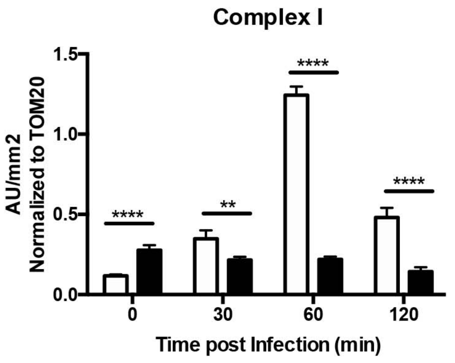

Supplemental Figure 1: Young and aged macrophages were cultured with *S. pneumoniae* (MOI=25). Mitochondria were isolated from young and aged macrophages at select time points post infection and protein expression was assessed using Total OXPHOS antibody cocktail. The blot was stripped and re-probed for TOM20 expression to confirm equivalent protein input between lanes. Full-length gels are included in Supplemental Figure 1 for comparison. Densitometry analysis for these gels is also included. Similar results were obtained from at least three independent experiments.

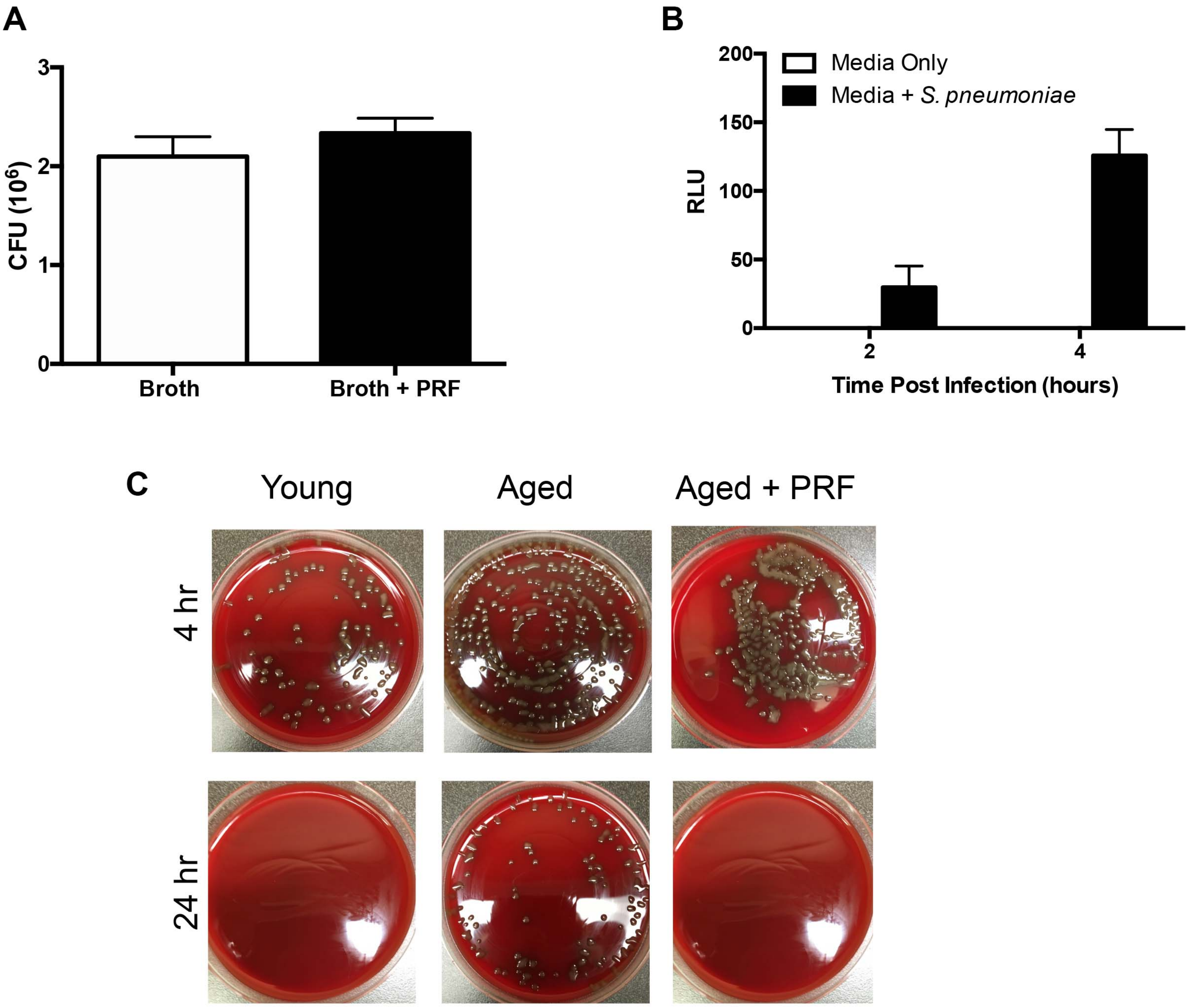

Supplemental Figure 2: *S. pneumoniae* was grown overnight on 10% sheep blood agar plates overnight. (A) An inoculating loop of bacteria was cultured in Todd Hewitt broth containing 2% yeast extract in the absence or presence of pirfenidone (500μg/ml) for 4 hours. Bacterial cultures were centrifuged for 15,000 x g for 1 minute and serial dilutions in broth were performed prior to plating. (B) Non-cellular H_2O_2 quantification of media alone or media containing *S. pneumoniae*. (C) Young, aged, and pirfendione (PRF) pre-treated aged macrophages were cultured with *S. pneumoniae*. Bacterial growth was assessed at 4 and 24 hours post culture. Similar results were obtained from at least two independent experiments.

A

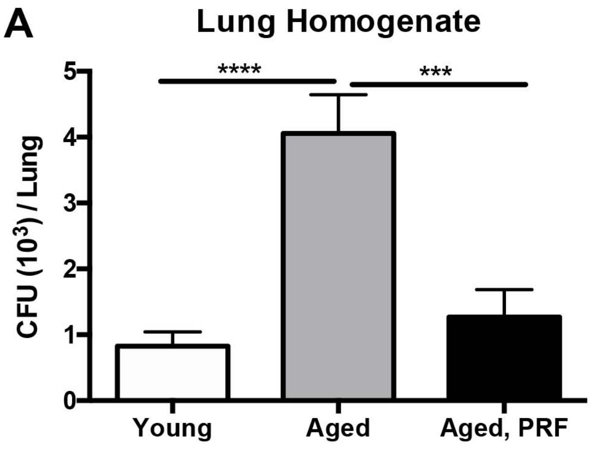

B

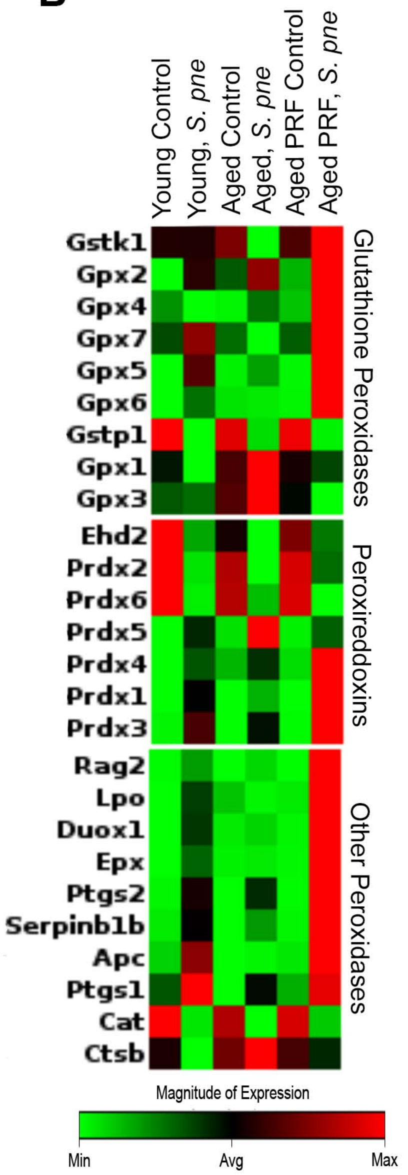

		**Young Lung, *S. pne***		**Aged Lung, *S. pne***		**Aged PRF Lung, *S. pne***	
**GENE SYMBOL**	**GENBANK**	**Fold Regulation (Young Control)**	**SD**	**Fold Regulation (Aged Control)**	**SD**	**Fold Regulation (Aged PRF Control)**	**SD**
Gstk1	NM_029555	1.644	0.509	0.773	0.286	1.239	0.383
Gpx2	NM_030677	1.057	0.263	1.635	0.439	1.214	0.020
Gpx4	NM_008162	0.8235	0.573	1.274	0.107	1.112	0.019
Gpx7	NM_024198	1.729	0.425	0.392	0.114	1.853	0.236
Gpx5	NM_010343	6.588	0.322	3.034	0.239	8.282	0.025
Gpx6	NM_145451	3.249	0.233	0.959	0.347	9.317	3.753
Gstp1	NM_013541	0.243	0.31	0.384	0.121	0.223	0.426
Gpx1	NM_008160	0.582	0.2	1.311	0.35	0.6285	0.335
Gpx3	NM_008161	0.721	0.996	1.248	0.072	0.547	0.038
Ehd2	NM_153068	0.482	0.693	0.422	0.053	0.493	0.037
Prdx2	NM_011563	0.503	0.151	0.641	0.044	0.521	0.125
Prdx6	NM_007453	0.271	0.123	0.45	0.293	0.213	0.021
Prdx5	NM_012021	1.717	0.182	4.141	0.263	1.248	0.188
Prdx4	NM_016764	0.979	0.118	1.375	0.097	1.274	0.217
Prdx1	NM_011034	3.116	0.721	2.079	0.29	4.626	0.021
Prdx3	NM_007452	1.328	0.237	1.602	0.33	1.385	0.031
Rag2	NM_009020	11.958	0.333	7.061	0.146	50.562	16.250
Lpo	NM_080420	2.203	0.294	0.461	0.158	4.626	1.299
Duox1	NM_001099297	5.205	0.448	1.827	0.116	10.928	1.950
Epx	NM_007946	5.098	0.704	1.394	0.048	13.642	1.810
Ptgs2	NM_011198	2.867	0.359	3.182	0.016	3.918	0.253
Serpinb1b	NM_173052	5.351	0.227	3.555	0.174	8.456	0.450
Apc	NM_007462	1.635	0.111	1.14	0.106	1.613	0.575
Ptgs1	NM_008969	1.464	0.669	1.515	0.038	1.231	0.151
Cat	NM_009804	0.318	0.575	0.381	0.179	0.305	1.083
Ctsb	NM_007798	0.619	0.437	1.231	0.185	0.646	0.613

Supplemental Figure 3: Young and aged untreated or pretreated with pirfenidone (PRF, 280mg/kg) mice were infected with *S. pneumoniae* ($1x10^6$ CFU). Lung tissue was collected at 24 hours post infection. (A) Serial dilutions of lung homogenates were plated on blood agar plates and CFU was quantified. (B) RNA was isolated and gene expression was assessed using Mouse Oxidative Stress PAMM-065ZA PCR arrays.
